# Socio-demographic and clinical characteristics of the perpetrators of sexual assaults assessed at the psychiatric department of Mahdia

**DOI:** 10.1192/j.eurpsy.2022.1553

**Published:** 2022-09-01

**Authors:** M. Kacem, W. Bouali, O. Charaa, N. Faouel, L. Zarrouk

**Affiliations:** University Hospital of Mahdia, Tunisia., Psychiatry, mahdia, Tunisia

**Keywords:** expertise, forensic psychiatry, SEXUAL ASSAULT, Indecent assault

## Abstract

**Introduction:**

Sexual assault constitutes a major problem in Tunisian society. There is no definitive typology of the characteristics of those who sexually assault. The great diversity of sexual assault behaviors and the different underlying motivations do not allow us to describe a typical profile of the sexual assailant. There may be cognitive, personality trait, lifestyle, and pathway distortions involved in the etiology and maintenance of deviant sexual behaviors.

**Objectives:**

To establish the socio-demographic and clinical profile of the perpetrators of sexual assault appraised in the psychiatric service of Mahdia.

**Methods:**

This is a descriptive retrospective file-based study on all subjects assessed at the Taher Sfar Mahdia psychiatric department for sexual assault during the period from January 01, 2010 to December 31, 2020.

**Results:**

Our sample consisted of 18 interviewed subjects. The median age was 40 years with extremes of age of the accused ranging from 30 to 61 years. The entire population is male. He was essentially of average socio-economic level. A psychiatric diagnosis was retained in 50% of the perpetrators of sexual assault: bipolar disorder (27.7%), schizophrenia (11.1%), antisocial type personality disorders (5.5%) and mental retardation (5.5%). Indecent assault was the most common assault followed by rape. The minors were victims in 33.3% of the cases Among those arrested, 72% were considered responsible for their acts and only one is considered irresponsible.

**Conclusions:**

The studies having focused on the characteristics of the sexual aggressors concluded with a profile of the young man, single and badly inserted which does not constitute in any case a typical profile.

**Disclosure:**

No significant relationships.

